# Design of a care pathway for pharmacy-based PrEP delivery in Kenya: results from a collaborative stakeholder consultation

**DOI:** 10.1186/s12913-020-05898-9

**Published:** 2020-11-12

**Authors:** Katrina F. Ortblad, Peter Mogere, Stephanie Roche, Kevin Kamolloh, Josephine Odoyo, Elizabeth Irungu, Nelly R. Mugo, Jillian Pintye, Jared M. Baeten, Elizabeth Bukusi, Kenneth Ngure, on Behalf of Stakeholders for Pharmacy-based PrEP Delivery in Kenya Consultation, Binti Tsala, Nancy Cherutich, Mary Mugambi, Abdullahi Sheikh, Mariah Mensah, Helgar Musyoki, Benjamin Mbugua, Louis Somoni, Elizabeth Gitau, Faith Tonui, Mercy Mutonyi, Jeffery Walimbwa, George Mugendi, Daniel Were, Victoria Wanjohi, Judy Kariuki, Nancy Abuya, Victoria Liaka, Beatrice Wachira, Moraa Kiangoi, Peter Mugo, Elyse Tung, Renee Heffron, Pamela Kohler, Andy Stergachis

**Affiliations:** 1 Department of Global Health, International Clinical Research Center, University of Washington, 908 Jefferson St, 12th floor, Seattle, WA 98104 USA; 2 Partners in Health and Research Development, Thika, Kenya; 3 Centre for Microbiology Research, Kenya Medical Research Institute, Nairobi, Kenya; 4 Sexual Reproductive and Adolescent Child Health Research Program, Kenya Medical Research Institute, Nairobi, Kenya; 5 School of Nursing, University of Washington, Seattle, USA; 6 Department of Epidemiology, University of Washington, Seattle, USA; 7 Department of Medicine, University of Washington, Seattle, USA; 8 Department of Obstetrics and Gynecology, University of Washington, Seattle, USA; 9 Department of Community Health, Jomo Kenyatta University of Agriculture and Technology, Nairobi, Kenya

**Keywords:** PrEP, HIV prevention, Pharmacy care, Kenya, Stakeholders, Implementation science

## Abstract

**Introduction:**

In Kenya, pre-exposure prophylaxis (PrEP) for HIV prevention is almost exclusively delivered at HIV clinics. Developing novel PrEP delivery models is important for increasing the reach of PrEP. Delivery of PrEP through pharmacies is one approach utilized in the US to improve accessibility. Retail pharmacies are commonly used as a first-line access point for medical care in Kenya, but have not been utilized for PrEP delivery. We conducted a collaborative consultative meeting of stakeholders to develop a care pathway for pharmacy-based PrEP delivery in Kenya.

**Methods:**

In January 2020, we held a one-day meeting in Nairobi with 36 stakeholders from PrEP regulatory, professional, healthcare service delivery, civil society, and research organizations. Attendees reviewed a theory of change model, results from formative qualitative research with pharmacy providers and clients, and anticipated core components of pharmacy-based PrEP delivery: counseling, HIV testing, prescribing, and dispensing. Stakeholders participated in small and large group discussions to identify potential challenges and solutions. We synthesized the key findings from these discussions.

**Results:**

Stakeholders were enthusiastic about a model for pharmacy-based PrEP delivery. Potential challenges identified included insufficient pharmacy provider knowledge and skills, regulatory hurdles to providing affordable HIV testing at pharmacies, and undefined pathways for PrEP procurement. Potential solutions identified included having pharmacy providers complete the Kenya Ministry of Health-approved PrEP training, use of a PrEP prescribing checklist with remote clinician oversight and provider-assisted HIV self-testing, and having the government provide PrEP and HIV self-testing kits to pharmacies during a pilot test. A care pathway was developed over the course of the meeting.

**Conclusions:**

PrEP delivery stakeholders in Kenya were strongly supportive of developing and testing a model for pharmacy-based PrEP delivery to increase PrEP access. We collaboratively developed a care pathway for pilot testing that has the potential to expand PrEP delivery options in Kenya and other similar settings.

**Supplementary Information:**

The online version contains supplementary material available at 10.1186/s12913-020-05898-9.

## Introduction

Maximizing access and reaching at-risk populations are key priorities for optimizing the public health impact of pre-exposure prophylaxis (PrEP) for HIV prevention [1, 2]. PrEP is highly effective and safe when taken as prescribed [3–6]. In sub-Saharan Africa, PrEP is being added to an already-stretched public health infrastructure, and the ability of the health systems to maximize PrEP access will necessitate finding novel delivery strategies [7]. In feasibility evaluations of PrEP in sub-Saharan Africa, major barriers to clinic-based PrEP delivery include stigma, long waiting times, the costs of staffing, and healthcare providers’ unfamiliarity with delivering prevention interventions [8–10].

In Kenya and many other resource-constrained countries, retail pharmacies fill an important gap in the health care system, providing access to treatment of urgent conditions (e.g., evaluation and medication for sexually transmitted infections [STIs]), monitoring of chronic conditions (e.g., blood pressure and diabetes), point-of-care testing (e.g., for malaria and HIV), and preventative care (e.g., contraception) [11–16]. Pharmacy-based care has many attributes that may be desirable for potential PrEP users, including convenience (as pharmacies outnumber clinics and have shorter waiting times), anonymity (compared to seeking PrEP at a public HIV care center), and engagement (which may be greater for a preventative service at a pharmacy than at a clinic that prioritizes treating patients with existing health conditions) [17–19]. Pharmacies can offer free, subsidized, or fully fee-for-service care, and paying for a service could result in greater sustained consumer engagement. The core components of PrEP – including HIV testing, adherence and risk reduction counseling, assessment of side effects, and provision of refills [20, 21] – are all within the scope of practice for pharmacy professionals [22, 23]. In the US, one model has demonstrated that PrEP can be provided completely by pharmacists, with oversight by a remote physician [24, 25].

To develop a model for pharmacy-based PrEP delivery for pilot testing in Kenya, we convened a meeting of stakeholders with diverse professional backgrounds and roles in PrEP delivery to collaboratively design a pathway that aligns with Kenya’s national PrEP and HIV prevention guidelines [21, 26]. In implementation science, the process of moving from formative research to the development of a model for testing is seldom detailed in the literature. This paper aims to document this process and present a collaboratively-developed model for pharmacy-based PrEP delivery that will be refined and tested before being scaled in Kenya and other similar settings.

## Methods

### Setting

In Kenya, PrEP is currently being delivered at most health facilities. In 2019, nearly 2000 public HIV clinics in Kenya were offering PrEP and approximately 63,000 individuals were on PrEP [27], with over half of them at public HIV clinics [28]. In 2019, there were 6375 registered pharmacies in Kenya: 5239 retail, 437 wholesale, and 799 hospital pharmacies [29]. The retail pharmacies we are focusing on in this study are primarily privately owned and staffed by pharmaceutical technologists. The Kenyan Ministry of Health (MOH) recently collaborated with private retail pharmacies to deliver HIV self-tests and facilitate linkage to care support and thus has established relationships with retail pharmacies across the country [30, 31].

### Stakeholder selection

We convened a consultation of individuals from different regulatory, professional, healthcare service delivery, civil society, and research organizations in Kenya (Appendix I). We included individuals from diverse professional backgrounds who had different roles in PrEP delivery so that we could develop the most comprehensive care pathway that included all the appropriate details for pharmacy-based PrEP delivery (in regards to testing, counseling, and record keeping), fit within existing Kenya national PrEP guidelines, and was acceptable to both implementers and regulators.

### Meeting activities

The one-day stakeholder meeting in Nairobi, Kenya featured presentations from stakeholders and researchers, as well as small and large group discussions. Two facilitators (EB, KN) with expertise in PrEP and implementation research oversaw the meeting. The meeting — including group discussions — was audio-recorded for purposes of note-taking.

Representatives from Kenya’s National AIDS & STI Control Program (NASCOP) and Pharmacy & Poisons Board (PPB) opened the meeting with presentations on the role of PrEP in Kenya’s national HIV prevention plan. Next, members of our research team presented a theory of change for how pharmacy-based PrEP delivery may overcome barriers to PrEP access and delivery and outlined the anticipated core components of pharmacy-based PrEP delivery in Kenya (i.e., counseling, HIV testing, prescribing, dispensing, oversight/referral). A mock care pathway for the delivery of these components was presented, based on a pharmacy-based PrEP delivery model ongoing in the US [24].

Following presentations, stakeholders were divided into three small groups to discuss the potential for pharmacy-based PrEP delivery to address the barriers to PrEP access and delivery presented in the theory of change model. Stakeholders were grouped purposefully to equally distribute different perspectives (e.g., pharmacy professionals, laboratory technologists, clinicians, researchers, governmental officials) across the groups. To help guide discussion, each group received worksheets (organized by the anticipated core components of pharmacy-based PrEP delivery) on current practices and potential barriers to pharmacy-based PrEP delivery (Appendix II) and potential solutions and regulatory requirements needed to address identified barriers (Appendix III). After completion of each worksheet, the small groups shared their discussion points with the larger group. After completion of the small and large group discussions, the facilitators summarized the key points that emerged during the discussions and presented an updated pharmacy-based PrEP delivery care pathway, which was interrogated by group consensus for its various proposed components.

We received ethical approval to conduct formative research, including engagement with stakeholders during a one-day meeting, from institutional review boards at the Kenya Medical Research Institute and University of Washington.

## Results

### Introductory statements

Both NASCOP and PPB gave introductory statements that were strongly supportive of developing models for pharmacy-based PrEP delivery in Kenya. The organizations acknowledged the limitations of clinic-based PrEP delivery and stressed the need for evidence that alternative models of PrEP delivery work. Both organizations emphasized their commitment to working with researchers to support the development of such models and outlined key questions related to the development of pharmacy-based PrEP delivery. Their questions centered around the minimum criteria pharmacies should meet to deliver PrEP, capacity-building needs among pharmacy providers,acceptable modalities of PrEP delivery at pharmacies (e.g., initiation at pharmacies and continuation at HIV clinics, initiation at HIV clinics and refills at pharmacies, etc.), data for tracking pharmacy-based PrEP delivery, and the role of NASCOP and PPB in the process.

### Theory of change & anticipated core components

**Fig. 1:**
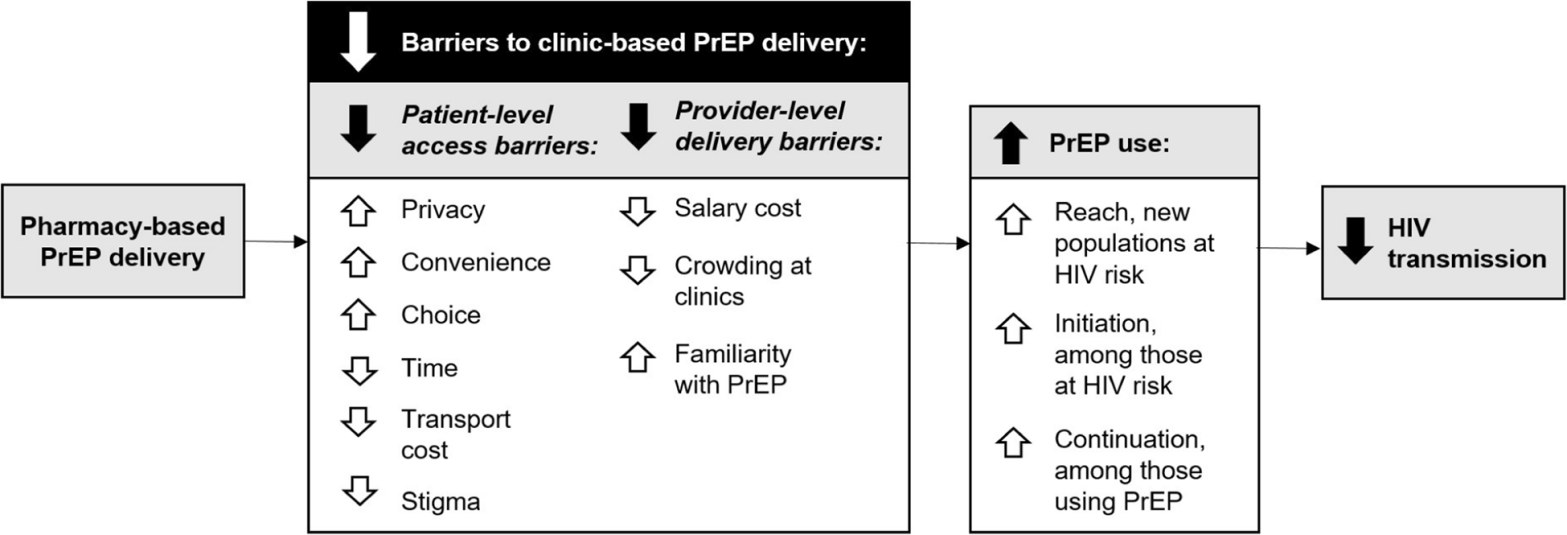
Theory of change for how pharmacy-based PrEP delivery may overcome barriers to clinic-based PrEP delivery in Kenya

In our theory of change model, Fig. 1, pharmacy-based PrEP delivery may overcome both patient-level and provider-level barriers to clinic-based PrEP delivery. Specifically, we anticipate that relative to clinic-based PrEP delivery, pharmacy-based PrEP delivery will increase patients’ privacy because individuals often come to pharmacies for a variety of reasons, convenience because retail pharmacies outnumber HIV clinics and have longer opening hours, and choice because individuals at HIV risk can select their preferred location for PrEP care. Additionally, we anticipate that pharmacy-based PrEP delivery will decrease the time patients spend waiting for PrEP care, the cost of traveling to a location where PrEP can be accessed, and the stigma associated with PrEP use (which is often amplified when individuals have to visit an HIV clinic for PrEP care). Pharmacy-based PrEP delivery may also decrease the salary costs of PrEP providers because retail pharmacy providers (i.e., pharmaceutical technologists) often make less than clinicians, decrease overcrowding at HIV clinics by moving care for healthy individuals to other settings, and increase the number of health providers that are familiar with and can provide PrEP care. Overall, we anticipate this novel model of PrEP delivery will increase the reach of PrEP to individuals at HIV risk who would never visit an HIV clinic for PrEP care, the total number of individuals initiating PrEP, and PrEP continuation (i.e., retention and adherence) among those using PrEP, ultimately decreasing overall HIV transmission in Kenya.

**Fig. 2:**
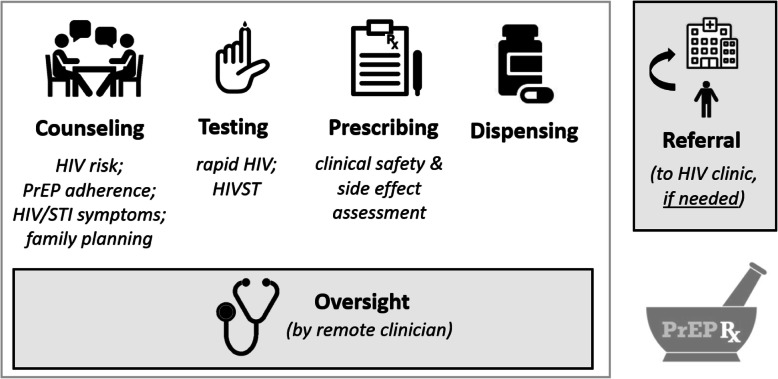
Anticipated core components of pharmacy-based PrEP delivery in Kenya. Source: Developed by the authors of this manuscript in collaboration with the Stakeholders for Pharmacy-based PrEP Delivery in Kenya (full permissions for image use received)

Following presentation of our theory of change model, we presented the anticipated core components of pharmacy-based PrEP initiation and refills: counseling, HIV testing, prescribing, and dispending, with oversight by a remote clinician and referral to a health facility when needed, Fig. 2.

### Challenges and solutions

During small and large group discussions, stakeholders identified a number of challenges and solutions, both for the pilot study of pharmacy-based PrEP delivery and for larger scale-up in Kenya. Findings from these discussions are summarized in Table 1. We subdivided the proposed solutions into short-term solutions that can be incorporated into the pilot study and long-term solutions that are geared more toward the future scale up of pharmacy-based PrEP delivery.

**Table 1:** Potential challenges and solutions to pharmacy-based PrEP delivery

Delivery component	Challenges	Solutions: Pilot	Solutions: Scale up
**Promoting pharmacy PrEP**	• Most existing promotional materials from suppliers. • Limitations on how pharmacies can advertise products/services. • Health facilities may be reluctant to refer client to pharmacy PrEP if trying to reach target PrEP numbers.	• Word-of-mouth promotion (e.g., PrEP providers at health facilities) • Tell clients seeking services indicating HIV risk-related behaviors (e.g., condoms, emergency contraception) about pharmacy PrEP • Display posters within the confines of the pharmacy.	• NASCOP works with pharmacies to create PrEP materials for display. • MOH national awareness campaign for pharmacy PrEP. • PPB revises advertisement restrictions for PrEP. • Pair pharmacies with health facility so pharmacy PrEP clients count toward the facility’s PrEP targets.
**HIV testing**	• No guidelines for rapid HIV testing at pharmacies, although ongoing. • Only select pharmacies currently providing assisted HIV self-testing. • Unclear if PrEP can be initiated based on an HIV self-test result. • Counterfeit HIV self-tests.	• Select pilot pharmacies certified to do assisted HIV self-testing. • Obtain special approval from regulatory agencies to offer HIV rapid testing at pilot pharmacies.	• MOH develops guidelines for HIV rapid testing at pharmacies (currently ~ 1/5th the price of self-tests at pharmacies). • MOH limits PrEP delivery to pharmacies certified to do assisted HIV self-testing (Obtain self-tests through KEMSA?). • PPB reclassifies HIV self-testing so treated like any other HIV test.
**Counseling**	• Pharmacy providers not trained on PrEP counseling. • No private space at some pharmacies. • Business approach of pharmacies, gets in the way of quality counseling. • Existing counseling prompted by clients, not pharmacy providers.	• Train pilot pharmacies on PrEP counseling using NASCOP guidelines and materials. • Private counseling space required for PrEP delivery at pharmacy.	• MOH implements pharmacy provider training requirement for PrEP delivery • MOH customizes NASCOP training to fit the retail pharmacy setting.
**Prescribing**	• Pharmacy providers not trained on how to (and not allowed to) prescribe PrEP. • What (cash) incentives do pharmacies have to provide PrEP if receive drug for free from NASCOP?	• Train pharmacy providers on how to prescribe using a checklist and remote clinician oversight. • Charge small consulting fee for PrEP counseling and dispensing. • Allow pharmacies to charge for HIV testing (necessary for prescription).	• PPB reschedules PrEP so that it can be sold without a prescription. • Pharmacy providers purchase PrEP from a generic manufacturer. • Pair pharmacies with CCC for oversight. • Remote PrEP clinicians (MOH supported?) for remote oversight.
**Dispensing**	• Retail pharmacies do not have MFL codes to: 1) acquire PrEP through KEMSA, and 2) report dispensing. • Current retail pharmacy records do not tend to track clients over time. • Some clients move between pharmacies. • Clients might not be able to afford 3-months PrEP at a time.	• Link pilot pharmacies with health facilities; have pharmacies use facility MFL code to obtain PrEP; then facilities reports the drugs dispensed. • Set up unique tracking system for pilot. • Have clients pay only for testing & a consulting/dispensing fee; PrEP drug free.	• Give pharmacies MFL codes. • Use system similar to diabetes for tracking prescriptions over time (e.g., “PrEP card”). • MOH provides free PrEP to pharmacies. • MOH establishes minimum criteria that pharmacy providers must meet to deliver PrEP (e.g., completion of NASCOP PrEP training).
**Oversight/ Referrals**	• Many retail pharmacies lack formal connections to health facilities. • Clinicians busy, often hard to reach. • Cost of oversight? Who pays? • Ethics – how do you know pharmacies will call clinicians when needed? • Who is the clinician?	• Link pharmacies with specific health facilities. • Have study-staff clinician on call. • Monitor the frequency of calls and record the content. • Create WhatsApp group for alternative mode of communication.	• NASCOP-supported PrEP clinician hotline? • Include cost of oversight in the consultation/dispensing fee client pays to pharmacy.
**Other**	• Some pharmacies only have one staff member working at a time. • Pharmacy providers may lack PrEP and HIV testing & counseling knowledge. • Pharmacy providers may discriminate against clients, especially marginalized populations (e.g., MSM, FSWs). • Currently, no regulations for pharmacy-based PrEP delivery.	• Pharmacy providers selected for this pilot will be trained on PrEP delivery and provided with a standardized checklist to walk them through PrEP prescribing. • Pharmacy providers will be connected to a remote PrEP clinician who can answer any questions they have and receive referrals of complex clients.	• MOH establishes minimum criteria (e.g., possession of a private consultation room, completion of NASCOP training) that pharmacy establishments and providers must meet to deliver PrEP. • MOH requires pharmacy-based PrEP providers to undergo a sensitization training on PrEP stigma/discrimination. • MOH establishes guidelines for pharmacy-based PrEP delivery, including any price regulation and accountability mechanisms (e.g., in cases of client mismanagement).

*Abbreviations*: *FSWs* female sex workers, *KEMSA* Kenya Medical Supplies Authority, *MFL* Master Facility List, *MSM* men who have sex with men, *MOH* Ministry of Health, *NASCOP* National AIDS and STI Control Program, *PPB* Pharmacy and Poisons Board, *PrEP* pre-exposure prophylaxis

Anticipated challenges primarily centered on pharmacy provider knowledge and skills to deliver PrEP. To ensure that the pharmacy providers in the pilot study have sufficient skills to deliver PrEP, stakeholders suggested training using existing NASCOP PrEP materials and guidelines. For future pharmacy PrEP scale up, stakeholders suggested that the Kenya MOH establish training requirements specifically for pharmacy providers and that NASCOP PrEP training materials be customized to fit the retail pharmacy setting. Stakeholders agreed that the pilot study should have pharmacy providers use a stakeholder-approved checklist to guide PrEP prescribing and a remote clinician available for consultation (via both the phone and WhatsApp). If this model is successful in a pilot setting, stakeholders expressed interest in testing this model at scale (potentially by pairing retail pharmacies with specific health facilities that can provide oversight, referral, and supply of PrEP medication) and, in the future, having PPB consider rescheduling PrEP medications so that is available without a prescription (pending scale-up findings).

Other anticipated challenges to pharmacy-based PrEP delivery included lack of guidelines for PrEP delivery in pharmacies, including whether and how pharmacies can advertise PrEP, test for HIV, procure and price PrEP, and document PrEP delivery. Stakeholders noted that, currently in Kenya, the PPB does not allow retail pharmacies to publicly advertise name-brand medicines. To attract PrEP clients during the pilot study, stakeholders advocated for pilot pharmacy providers to use word-of-mouth referral, offer PrEP to clients seeking services indicative of HIV risk behavior (e.g., emergency contraception, STI treatment), and obtain permission to display PrEP posters within the confines of the pharmacy. Stakeholders also suggested that, for future pharmacy-based PrEP scale-up, the PPB could revise pharmacy advertising restrictions to include a PrEP exemption, the MOH could implement a national awareness campaign, and NASCOP could create standard PrEP promotional materials for pharmacies.

With respect to HIV testing, stakeholders noted that retail pharmacies are not currently allowed to sell or perform rapid HIV tests but that they are allowed to sell and assist clients with HIV self-testing. For the pilot study, stakeholders felt that only provider-assisted HIV self-testing should be permissible, but that long term solutions for pharmacy-based PrEP scale-up could include developing MOH guidelines for the delivery of rapid HIV testing at pharmacies (including modifying permissions as to who can conduct rapid HIV tests).

With respect to PrEP procurement and pricing, NASCOP and MOH representatives committed to providing the PrEP drugs and HIV self-tests for free to the pharmacies in the pilot study and suggested that pilot pharmacies acquire their PrEP and HIV self-tests through nearby health facilities so that this inventory can be tracked using those facilities’ Master Facility List (MFL) codes. The cost of PrEP to pilot study participants would, therefore, be just a service fee for counseling, and dispensing, but not for the HIV self-test or PrEP drug itself. When asked how PrEP procurement and pricing might be handled in a scale-up situation, stakeholders felt that pharmacy providers could work with non-branded drug manufacturers to procure PrEP and HIV tests, or the MOH could consider giving retail pharmacies their own MFL codes for direct procurement through the Kenya Medical Supplies Agency (KEMSA).

### Care pathway for pharmacy-based PrEP delivery

**Fig. 3:**
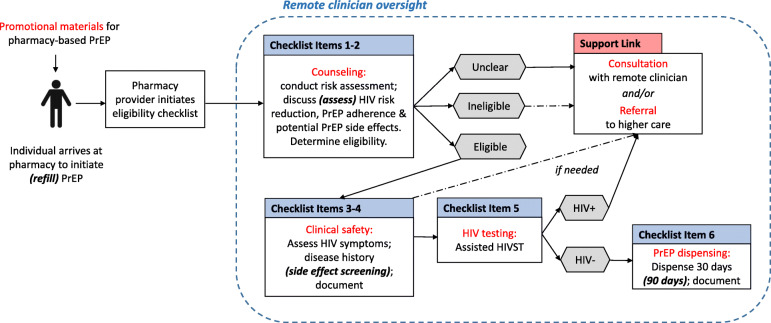
Proposed care pathway for pharmacy-based PrEP delivery pilot testing. Source: Developed by the authors of this manuscript in collaboration with the Stakeholders for Pharmacy-based PrEP Delivery in Kenya (full permissions for image use received)

Based on stakeholder feedback and consensus, we revised and finalized the care pathway for pharmacy-based PrEP delivery to pilot test, Fig. 3. We organized this pathway using a PrEP prescribing checklist (Table 2, more detailed form in Appendix III), which includes HIV risk assessment (using the MOH-approved Rapid Assessment Screening Tool), counseling (on HIV risk reduction and PrEP adherence), clinical safety assessment (including screening for HIV symptoms, disease history, and potential PrEP side effects), HIV testing, and PrEP dispensing. This care pathway for pharmacy-based PrEP delivery is designed only for healthy individuals at HIV risk with low likelihood of any drug contraindications. Thus, is it important that pharmacy providers delivering PrEP are trained on approaches to screen potential and continuing PrEP clients for pre-existing (e.g., diabetes, kidney disease) or emerging (e.g., pregnancy, side effects associated with PrEP use) health conditions that might impact safe PrEP use (using the described checklist) and refer those with any identified conditions to a public HIV clinic for continued PrEP care. If a pharmacy PrEP provider has any concerns or questions regarding the health or eligibility of a (potential) PrEP client, he or she can consult a remote clinician via phone or SMS.

**Table 2:** Prescribing checklist for pharmacy-based PrEP delivery

Checklist item	
**1. HIV risk:** at risk (using Rapid Assessment Screening Tool)	
**2. Counseling:** completed (e.g., risk reduction & PrEP adherence)	
**3. HIV symptoms:** none	
**4. Clinical safety & side effect assessment:** confirmed (e.g., no kidney disease)	
**5. HIV status:** negative (confirmed with testing)	
**6. Records:** completed	

## Discussion

This collaborative meeting, including a wide cross-section of stakeholders, aimed to spearhead the first national effort to create a formal pathway for pharmacy-based PrEP delivery that bridges the public and private sectors [26, 28]. This stakeholder meeting represents an important initial step by establishing consensus around a pharmacy-based PrEP delivery model to pilot test and identifying considerations for future scale-up, in Kenya and similar settings. If it can be implemented successfully, Kenya’s model for pharmacy-based PrEP delivery could trail blaze a path for other countries in sub-Saharan Africa, thus providing a template for other ministries of health to adapt. Here, we describe the implications of the stakeholder meeting for our pilot study, which is slated to begin in the third quarter of 2020.

In its 2017 framework for PrEP implementation, the Kenya MOH identified research on the feasibility of integrating PrEP into pharmacies as “high priority” [26]. At this stakeholder meeting, the MOH confirmed its commitment to this objective by expressing support for this pharmacy-based PrEP delivery research and working with other key stakeholders to collaboratively develop a care pathway for pilot testing. A major strength of the resulting pathway is that it is grounded in expert knowledge of the Kenyan PrEP landscape. As such, the pathway leverages existing resources (e.g., NASCOP PrEP training materials) and incorporates innovative work-arounds to potential barriers (e.g., a novel checklist to help pharmacy providers establish PrEP client eligibility).

Expanding the scope of retail pharmacy practice to include interventions traditionally delivered at healthcare clinics is not new for Kenya. For years, retail pharmacies have delivered family planning methods (e.g., condoms, oral contraception, emergency contraception and, more recently, injectable contraception) that are available for free at public family planning clinics [14]. The pharmacy-based PrEP delivery model we aim to pilot test represents a shift toward de-medicalizing PrEP in the sense that some tasks previously reserved to clinicians (e.g., writing PrEP prescriptions) will be expanded to a new cadre: pharmacy providers. If pharmacy providers prove able to adequately assess PrEP eligibility, adherence, and side effects, and some PrEP clients opt to get PrEP at pharmacies rather than healthcare facilities, this could potentially help decongest health facilities while also satisfying PrEP clients who prefer pharmacy-based care and might face discrimination accessing PrEP at health facilities.

Similarly, this model — if successful — may prompt the Kenya MOH to reconsider HIV testing guidelines. According to guidelines by Kenya Medical Laboratory Technicians and Technologists Board, only trained laboratory technologist and technicians are legally allowed to conduct rapid provider-completed HIV testing in Kenya. Under these guidelines, the ongoing rapid HIV testing being conducted by nurses at health facilities and retail pharmacy providers is not permissible. For these reasons, we limited HIV testing in our pharmacy-based PrEP delivery pilot study to provider-assisted HIV self-testing, which was made permissible when HIV self-tests were introduced in Kenya in May 2017 [30]. There exists, however, a high demand for HIV testing at retail pharmacies, and among pharmacies that currently provide both rapid HIV tests and self-tests, rapid tests are roughly one-third the cost of self-tests. Revising guidelines on who is legally allowed to provide rapid HIV testing might be something for Kenyan stakeholders to consider in the future to expand access to HIV testing services and decrease the potential cost of pharmacy-based PrEP delivery.

Documentation of PrEP dispensing was another key concern raised during the stakeholder meeting because implementing partners at the MOH facilities set PrEP targets for health facilities that could potentially disincentivize facilities from referring PrEP clients to retail pharmacies. For the pilot study, it is important that PrEP dispensing records complement — and do not compete with — facilities’ PrEP dispensing targets. For this reason, the PrEP dispensed at pilot pharmacies was recommended to be under the MFL code of a nearby health facility so that it can count toward that facility’s target PrEP numbers. Accurate completion of NASCOP’s PrEP dispensing records (including the drugs that will be donated to the pilot pharmacies) is important for future PrEP procurement, including continued support from international organizations such as the United States President’s Emergency Plan for AIDS Relief, United States Agency for International Development, Global Fund, and others. A number of electronic drug dispensing tools have already been developed by the Pharmaceutical Society of Kenya and the Clinton Health Access Initiative for use in the Kenyan context, so we plan on modify existing tools to record pharmacy-based PrEP delivery in our pilot study.

This study has some limitations that are important to note. First, the pharmacy PrEP stakeholders who attended this Nairobi-based meeting were a highly selective group of individuals who had contributed, in some way, to the recent introduction and scale up of PrEP in Kenya. While an alternative group of stakeholders may have designed a different care pathway for pharmacy-based PrEP delivery, we believe the experiences of the stakeholders in this group helped informed the design of a care pathway with the greatest likelihood of success, particularly for the setting. Second, the pharmacy-based PrEP care pathway designed from this collaborative process does not include creatinine testing for PrEP initiation. In Kenya, creatinine testing is not formally required for starting PrEP if doing so would limit initiation [21]. Additionally, programmatic data suggest that < 3% of individuals initiating PrEP at public HIV clinics receive creatinine testing [32]. In individuals with no risk factors, renal compromise with PrEP use is an extremely rare outcome [33]; which is why, in our proposed care pathway, only healthy individuals with no pre-existing or emerging health conditions are eligible for PrEP initiation or continuation at retail pharmacies. Third, the care pathway for pharmacy-based PrEP delivery developed in this study has not yet been tested in Kenya. Before this model of PrEP delivery is scaled in Kenya, model weak points should identified and refined through pilot testing (forthcoming) and the effect of pharmacy-based versus facility-based PrEP delivery on PrEP uptake and continuation should be measured through a randomized trial. Finally, because Kenya was one of the first countries to deliver PrEP in sub-Saharan Africa, it has a more mature PrEP delivery program than other sub-Saharan African countries. For this reason, the pharmacy-based PrEP delivery care pathway developed by Kenyan stakeholders might have limited generalizability to settings outside Kenya and thus should be adapted to and tested in new settings before being implemented.

## Conclusions

Through a collaborative stakeholder consultation process, we were able to successfully develop a care pathway for pilot testing of pharmacy-based PrEP delivery in Kenya. Despite the availability of highly effective HIV prevention interventions, high levels of population-level HIV incidence persist in many sub-Saharan African settings [1]. Novel strategies to expand the reach of HIV prevention services to those most at HIV risk remain needed, including moving HIV prevention services outside of clinical settings (e.g., pharmacies) to reach individuals that might never frequent health facilities for a multitude of reasons, including long wait times and HIV stigma [8–10]. In our consultation process, pharmacy PrEP stakeholders expressed strong enthusiasm and support for pharmacy-based PrEP delivery in Kenya [26, 28] and identified a number of challenges and potential solutions for implementing this novel model of PrEP delivery. Now generating evidence around the effectiveness of pharmacy-based PrEP on PrEP uptake and continuation is a necessary first step toward making this highly effective HIV prevention intervention accessible to those who need it.

## Supplementary Information

**Additional file 1:** **Appendix I.** Stakeholder organizations in attendance at the January 2020 meeting.

**Additional file 2:** **Appendix II.** Pharmacy-based PrEP delivery – Worksheet #1.

**Additional file 3:** **Appendix III.** Pharmacy-based PrEP delivery – Worksheet #2.

**Additional file 4:** **Appendix IV.** Pharmacy PrEP prescribing Checklist.

## Data Availability

We have included the worksheets used by stakeholders as well as the PrEP prescribing checklist for pharmacy-based PrEP delivery as Appendices of this paper. Slides presented at the stakeholder meeting are available upon request.

## Abbreviations

AIDS: Acquired immunodeficiency virus
CHAI: Clinton Health Access Initiative
HIV: Human immunodeficiency virus
KMLTTB: Kenya Medical Laboratory Technicians and Technologists Board
KEMSA: Kenya Medical Supplies Agency
MFL: Master facility list
MOH: Ministry of Health
NASCOP: National IADS & STI Control Program
PEPFAR: President’s Emergency Plan for AIDS Relief
PPB: Pharmacy and Poisons Board
PrEP: Pre-exposure prophylaxis
PSK: Pharmaceutical Society of Kenya
STIs: Sexually transmitted infections
USAID: United States Agency for International Development
