# Supplementary material for: Design of a care pathway for pharmacy-based PrEP delivery in Kenya: results from a collaborative stakeholder consultation
Source: BMC Health Serv Res. 2020 Nov 12;20:1034. doi: 10.1186/s12913-020-05898-9 (PMC7661206; doi:10.1186/s12913-020-05898-9)
Supplement: Supplementary file 1 — Additional file 1: Appendix I. Stakeholder organizations in attendance at the January 2020 meeting. [file 12913_2020_5898_MOESM1_ESM.pdf]

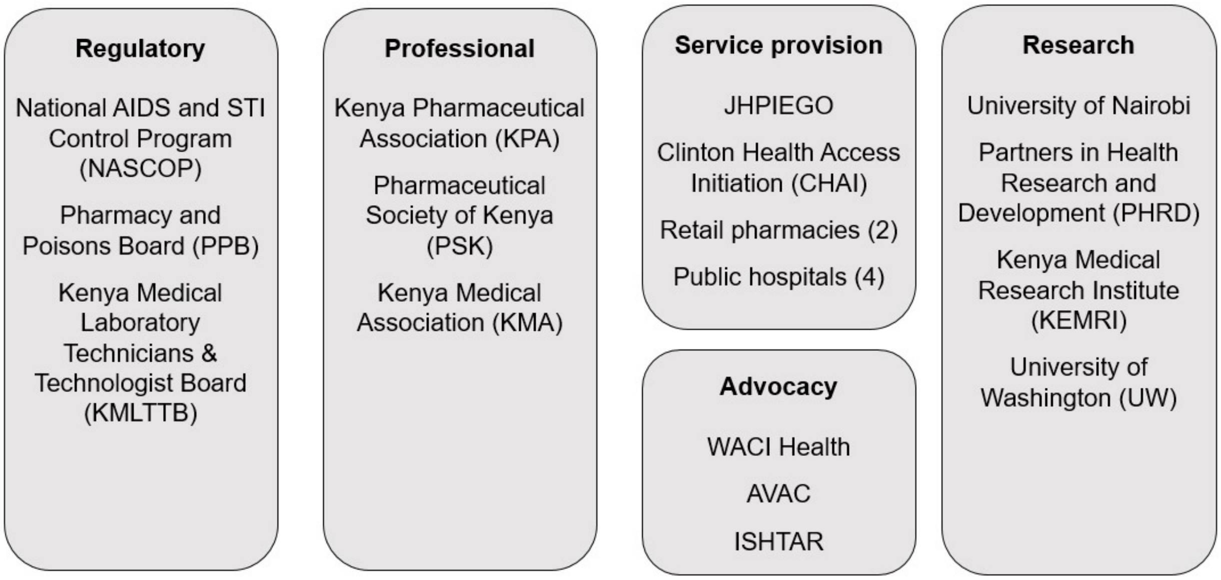

Appendix I. Stakeholder organizations in attendance at the January 2020 meeting

The retail pharmacies in attendance included Huruma Pharmacy and Leleshwa Pharmacy. The hospitals in attendance included Thika Level 5 Hospital, Mbagathi Hospital, Ruiru Hospital, and Karatina Hospital.
