# Supplementary material for: Design of a care pathway for pharmacy-based PrEP delivery in Kenya: results from a collaborative stakeholder consultation
Source: BMC Health Serv Res. 2020 Nov 12;20:1034. doi: 10.1186/s12913-020-05898-9 (PMC7661206; doi:10.1186/s12913-020-05898-9)
Supplement: Supplementary file 2 — Additional file 2: Appendix II. Pharmacy-based PrEP delivery – Worksheet #1. [file 12913_2020_5898_MOESM2_ESM.pdf]

Appendix II: Pharmacy-based PrEP delivery – Worksheet #1

Core component	Part 1: Current practice & barriers	
	Current practice (personnel/supplies/$$)	***Potential barriers***
**(Promotional materials)**<br>*Targeting HIV at-risk individuals (e.g., posters, pharmacy provider recommendation)*		
**Counseling**<br>*e.g., HIV risk reduction, importance of PrEP adherence, potential PrEP side effects, other?*		
**HIV testing**<br>*Prior to PrEP initiation and refill (includes counseling). Location of testing? Rapid HIV test or self-test?*		
**Prescribing**<br>*Written once clinical safety determined & checklist completed. Who? Records?*		
**Dispensing**<br>*After side effect assessment completed and everything on checklist ticked. Records?*		
**Oversight/Referrals**<br>*Includes remote clinician oversight; clinic referral; record keeping; reporting; etc.*		
