# Supplementary material for: Design of a care pathway for pharmacy-based PrEP delivery in Kenya: results from a collaborative stakeholder consultation
Source: BMC Health Serv Res. 2020 Nov 12;20:1034. doi: 10.1186/s12913-020-05898-9 (PMC7661206; doi:10.1186/s12913-020-05898-9)
Supplement: Supplementary file 3 — Additional file 3: Appendix III. Pharmacy-based PrEP delivery – Worksheet #2. [file 12913_2020_5898_MOESM3_ESM.pdf]

Appendix III: Pharmacy-based PrEP delivery – Worksheet #2

Core component	Part 2: Solutions & regulatory<br>*Potential solutions*	*Regulatory considerations*
**(Promotional materials)**<br>*Targeting HIV at-risk individuals (e.g., posters, pharmacy provider recommendation)*		
**Counseling**<br>*e.g., HIV risk reduction, importance of PrEP adherence, potential PrEP side effects, other?*		
**HIV testing**<br>*Prior to PrEP initiation and refill (includes counseling). Location of testing? Rapid HIV test or self-test?*		
**Prescribing**<br>*Written once clinical safety determined & checklist completed. Who? Records?*		
**Dispensing**<br>*After side effect assessment completed and everything on checklist ticked. Records?*		
**Oversight/Referrals**<br>*Includes remote clinician oversight; clinic referral; record keeping; reporting; etc.*		
