# Supplementary material for: Design of a care pathway for pharmacy-based PrEP delivery in Kenya: results from a collaborative stakeholder consultation
Source: BMC Health Serv Res. 2020 Nov 12;20:1034. doi: 10.1186/s12913-020-05898-9 (PMC7661206; doi:10.1186/s12913-020-05898-9)
Supplement: Supplementary file 4 — Additional file 4: Appendix IV. Pharmacy PrEP prescribing Checklist. [file 12913_2020_5898_MOESM4_ESM.pdf]

Appendix IV. Pharmacy PrEP prescribing Checklist

Pharmacy: ____________________ Provider: ____________________ Qualifications: ______________ MFL code: ___ ___ ___ ___ ___

CLIENT PROFILE:
Unique client record number: ___ ___ ___ ___ ___/ ___ ___ ___ ___/ ___ ___ ___ ___ ___

Visit date: dd / mm / yyyy
Initiating PrEP: ☐ Yes ☐ No

Name: First ________________ Middle ________________ Last ________________ Telephone no: ____________
Alien/National ID/passport/Birth Cert No: ________________ Came to pharmacy for: ______________________________

PrEP initiation ONLY:
Sex: ☐ Male ☐ Female Date of birth: dd / mm / yyyy Age (years): ____ ____ If age <19, attends school: ☐ Yes ☐ No
Marital status: ☐ Never married ☐ Cohabiting ☐ Married monogamous ☐ Married polygamous ☐ Separated/divorced ☐ Widowed
Population type: ☐ Gen Population ☐ Discordant couple ☐ Key Population (Specify) → ☐ MSM ☐ MSW ☐ FSW ☐ PWID

[If transferred in:] PrEP start date: dd / mm / yyyy Regimen: ☐ TDF-FTC ☐ TDF ☐ TDF-3TC
Facility transferred from: ________________ MFL code: __ __ __ __ __ County: ________________

PrEP SCREENING:

Behavioral risk assessment: *Mark all that apply (past 6 months)*

Sex partner(s) is HIV+ AND: *not on ART, or On ART <6 months, or suspected poor ART adherence, or detectable viral load, or couple trying to conceive*	☐ Yes	☐ No
Sex partner(s) high risk & HIV status is unknown:	☐ Yes	☐ No
Has sex with >1 partner	☐ Yes	☐ No
Ongoing IPV/GBV	☐ Yes	☐ No
Transactional sex	☐ Yes	☐ No
Recent STI (past 6 months)	☐ Yes	☐ No
Recurrent use of post-exposure prophylaxis (PEP)	☐ Yes	☐ No
Recurrent sex under influence of alcohol/recreational drugs	☐ Yes	☐ No
Inconsistent or no condom use	☐ Yes	☐ No
Injection drug use with shared needles and/or syringes	☐ Yes	☐ No

Any Yes → ***At HIV risk; CONTINUE with PrEP initiation at pharmacy***

Counseling

Willing to start/continue PrEP:	☐ Yes	☐ No
Adherence counseling Done:	☐ Yes	☐ No
Side effect counseling Done:	☐ Yes	☐ No
Family planning counseling Done:	☐ Yes	☐ No

All Yes → ***CONTINUE with PrEP initiation at pharmacy***

Medical safety assessment

Signs & symptoms of acute HIV Infection:	☐ Yes	☐ No
Signs & symptoms of COVID-19 infection:	☐ Yes	☐ No
Liver disease:	☐ Yes	☐ No
Kidney disease:	☐ Yes	☐ No
Diabetes:	☐ Yes	☐ No
Other: ________________	☐ Yes	☐ No
[If female:] ☐ Pregnant or ☐ Breastfeeding:	☐ Yes	☐ No

Any Yes → ***DO NOT start PrEP; refer to remote clinician***

PrEP refills ONLY:

Reported PrEP side effects:	*Describe:* ________________ ________________ ________________	☐ Yes	☐ No
[if yes:] Side effects severe:		☐ Yes	☐ No

Yes → ***DO NOT start PrEP; refer to remote clinician***

HIV testing
HIV test result: ☐ Negative ☐ Positive ☐ Indeterminate [Retest]
[If retest:] HIV test result: ☐ Negative ☐ Positive ☐ Indeterminate

Positive / Indeterminate → ***DO NOT start PrEP; refer to remote clinician***

PrEP DISPENSING:
Regimen: ☐ TDF-FTC ☐ TDF ☐ TDF-3TC
# of months prescribed: ___ ___
Date of initiation: dd / mm / yyyy

Next appointment date: dd / mm / yyyy
Provider initials: ________

PrEP Rx
